# Spontaneous Migration of Polyethylene Molecule Sheathed inside Single-Walled Carbon Nanotube for Nano-Heat Pipe

**DOI:** 10.1038/srep26441

**Published:** 2016-05-23

**Authors:** Quanwen Liao, Zhichun Liu, Nuo Yang, Wei Liu

**Affiliations:** 1 School of Energy and Power Engineering, Huazhong University of Science and Technology (HUST), Wuhan 430074, People’s Republic of China

## Abstract

Development of nanoscale thermal exchanging devices is critical to thermal management in nanoscale. The spontaneous migration of polyethylene molecule sheathed inside single-walled carbon nanotube (SWCNT) are observed. And the multi-factor analysis of spontaneous migration about temperature, mass and potential barrier shows new features about motion mechanisms, and enriches the existing mass transport theory greatly. Here, based on this finding, we report a nano-heat pipe (NHP) composing of a SWCNT and a polyethylene molecule. Using molecular dynamics simulations, the heat exchanging coefficient can reach 450 WK^−1^ cm^−2^ at 500 K by NHP arrays with a quantity density of 7 × 10^13^ cm^−2^. This study will benefit the designs of NHP and other nanoscale mass transport devices.

Since their discovery, the possibility of applying carbon nanotubes (CNTs) in heat and mass transfer has been an intriguing prospect for these hollow nanostructures. Recently, there has been significant progress in using CNTs as various types of sensors^1^,^2^ and mass transport tubes^3^,^4^,^5^,^6^,^7^. Benefited from the powerful high-resolution transmission electron microscopy facilities for manipulations with materials with the nanometer scale precision, the nanothermometer^2^, electrical switches^2^, nanorobotic spot welding^8^, nanopipettes^9^, archival memory^10^, nanotube-based motors^11^ and seawater desalination^12^ have been proposed to fabricate nanoscale devices, based on the electric/thermal driven migrations of the melts sheathed inside CNTs. However, nano-heat pipe (NHP) taking the advantage of the spontaneous migrations of polyethylene (PE) molecules encapsulated into single-walled carbon nanotubes (SWCNTs) has been absent from the previous studies. These NHP devices are desired in nanoscale hotspots such as junctions and defects in integrated circuits, where the thermal stress is the primary cause of the their invalidations^13^. Moreover, there are several theoretical predictions^11^,^14^ about the random walk effect, but no experimental or numerical works has been done.

In the present study, the spontaneous migrations of PE molecules encapsulated into SWCNTs in the absence of artificial force fields (such as temperature gradients and electric biases) are reported. Moreover, the elucidations of the dominating migration mechanism are sought by molecular dynamics (MD) simulations using the large-scale atomic/molecular massively parallel simulator (LAMMPS) package developed by Sandia National Laboratories^15^. Our discoveries explain this random walk effect with multi-factors (such as temperature, mass and potential energy) and enrich the existing mass transport theory. Based on these findings, an individual SWCNT filled with a PE molecule is used as a NHP. The processes we describe in this work are of both fundamental and applied interest.

## Part 1: Motion Mechanism Analysis

### Models and Methods

**Figure 1:**
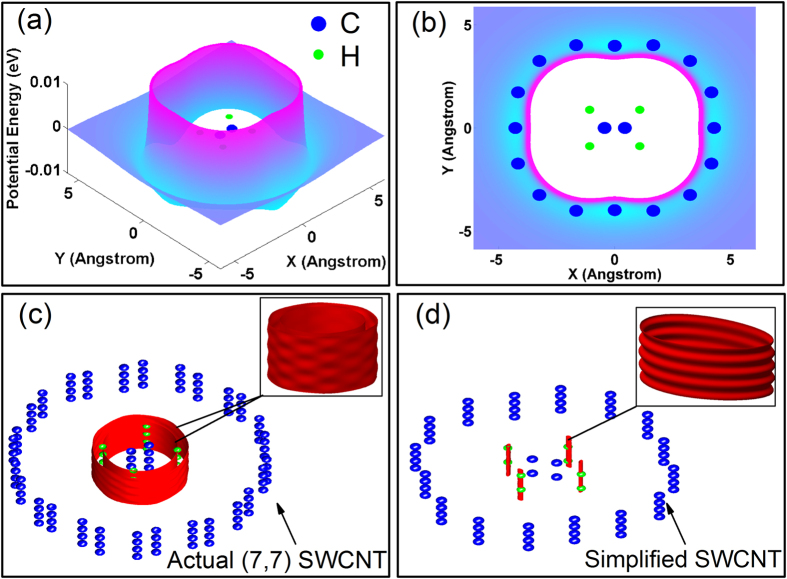
(**a**) The cross-sectional potential energy surface of PE. There are local minima at the light blue region. (**b**) The atoms of the SSWCNT are uniformly located at the local minimum region every 22.5°. (**c**) and (**d**) are the equipotential surface of an actual (7, 7) SWCNT and a SSWCNT interacting with the hydrogen atoms in PE, respectively.

A simplified model of SWCNT is applied to analyze the spontaneous migration as shown in Fig. 1(a,b). Figure 1(a) shows the potential energy on the cross section of PE. Figure 1(b) shows the adopted cross-sectional carbon atoms of simplified SWCNT (SSWCNT), which are evenly located in the minimum potential region of the PE molecule. The SSWCNT is obtained by replicating this carbon atom layer periodically in the longitudinal (*z*) directions. The longitudinal intervals between carbon atom layers are 1.27 Å that is consistent with the intervals between adjacent methylene (CH_2_) groups.

As the SSWCNT is deliberately chosen based upon minimum potential energy of PE, it has its specific roles in restricting PE in turn. Because the cross-sectional undulations of an actual SWCNT interfere with the longitudinal motions of PE molecules, we employ a SSWCNT whose equipotential surface shows quasi-1D periodic potential wells with undulations only in longitudinal direction, as shown in Fig. 1(d). Further, the hydrogen atoms are about located at the minimum potential regions, indicating that the artificial SSWCNT tends to suppress the cross-sectional movements of PE molecules, such as wiggle and torsion, compared to the actual SWCNT shown in Fig. 1(c). In summary, this SSWCNT highlights the main factors that are directly related to the longitudinal motions of the PE molecule in the tube, and enables us to explore these motions with ease.

Our simulating domain contains a frozen SSWCNT with a length of 20 nm and a PE molecule with a length of 4 nm. The atomic interaction of SSWCNT is described by a LJ potential with *ε* = 0 to lower the computational demands. Because their motion is relative, the frozen SSWCNT won’t make much difference to the results of PE molecule and make it easier to study the phenomenon. The atomic interaction of PE molecule is described by an adaptive intermolecular reactive empirical bond order (AIREBO) potential^16^ developed from the second-generation Brenner potential^17^. In addition, the atomic interactions between the PE molecule and SSWCNT are described by the LJ potential:


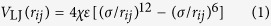


where the *ε* is the depth of the potential well; *χ* is coefficient of the potential well depth; *σ* is the finite distance at which the inter-particle potential is zero; *r*_*ij*_ is the interatomic distance. The parameters are *σ*_C-C_ = 3.4 Å, ε_C-C_ = 0.0028 eV, *σ*_C-H_ = 3.025 Å, and *ε*_C-C_ = 0.0021 eV; the C-C and C-H subscripts represent the carbon-carbon atomic interactions and the carbon-hydrogen atomic interactions, respectively. The cutoff distance for AIREBO and LJ potentials is 10.2 Å.

Equilibrium molecular dynamics (EMD) simulations are used to research the random walk effects. The periodic boundary conditions are applied in three directions, and cross-sectional (*x* and *y*) sizes of simulating boxes are large enough to avoid interactions between PE molecules. Initially, Nose-Hoover heat reservoir is used to equilibrate the PE molecule at desired temperature for 100 ps with the longitudinal linear momentum zeroed. Then, it is running in microcanonical ensemble for another 100 ps to eliminate the after-effects. Eventually, the simulations run in microcanonical ensemble for 22 ns without linear momentum zeroed, and the center of mass (COM) of the PE molecule are recorded during the latter 20 ns.

### Results and Discussions

For relatively well-studied electric driven mass transport, there are mainly three mechanisms: thermal evaporation, thermomigration, and electromigration^14^. However, little attention has been paid to the random walk effect of PE molecules in SWCNT. Generally, the random walk effect is attributed to the combination of the atomic oscillation and the longitudinal undulations of potential wells. Due to atomic oscillations, the roughness of equipotential surface provides longitudinally random force on each atom. The instantaneous fluctuations of resultant force could drive PE molecules to migrate spontaneously circumstantially. Therefore, the spontaneous migration is affected synthetically by atomic oscillation, roughness of equipotential surface and the size of PE molecule. By EMD the dependence of its motion characteristics upon temperature (*T*), length of PE molecule (*L*) and *χ* are studied with a SSWCNT. The *T*, *L* and *χ* are related to atomic oscillation, the size of PE molecule and roughness of equipotential surface respectively.

**Figure 2:**
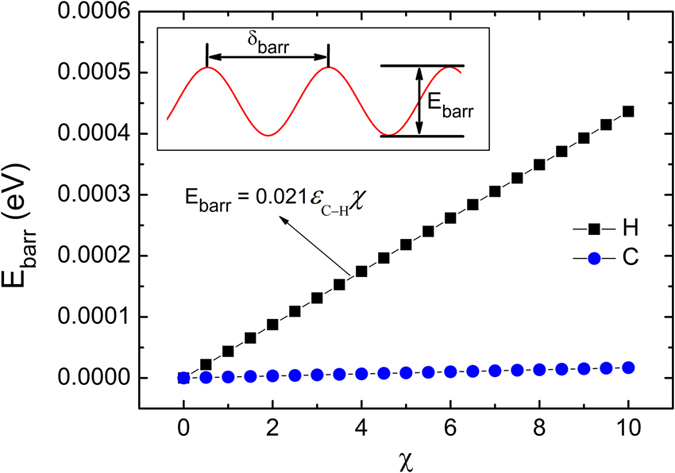
The barrier heights (*E*_barr_) per atom in PE molecule versus the coefficient of the depth of potential well (*χ*). There are directly linear relationship between the *E*_barr_ and *χ*. The insert shows the schematic potential energy curve in the SSWCNT along the longitudinal direction. The width of the periodical potential trap (*δ*_barr_) is 1.27 Å.

The roughness of equipotential surface can be evaluated by barrier height (*E*_barr_) which represents the energy to go over potential barrier. Figure 2 shows the relationship between barrier height and *χ* of carbon and hydrogen atoms in PE. Because carbon atoms are farther from SSWCNT than hydrogen atoms, the *E*_barr_ of carbon atom is only 3.8 percent that of hydrogen atom. Moreover, in PE the number of carbon atoms is half of the hydrogen atoms. So that the carbon atoms’ contribution is almost negligible in the total *E*_barr_. Based on this approximation, the activation barrier of a PE molecule approximates to


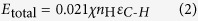


where *n*_H_ is the number of hydrogen atoms in a PE molecule. The width of the periodical potential trap (*δ*_barr_) is 1.27 Å, which is consistent with the intervals of carbon atom layers of SSWCNT.

**Figure 3:**
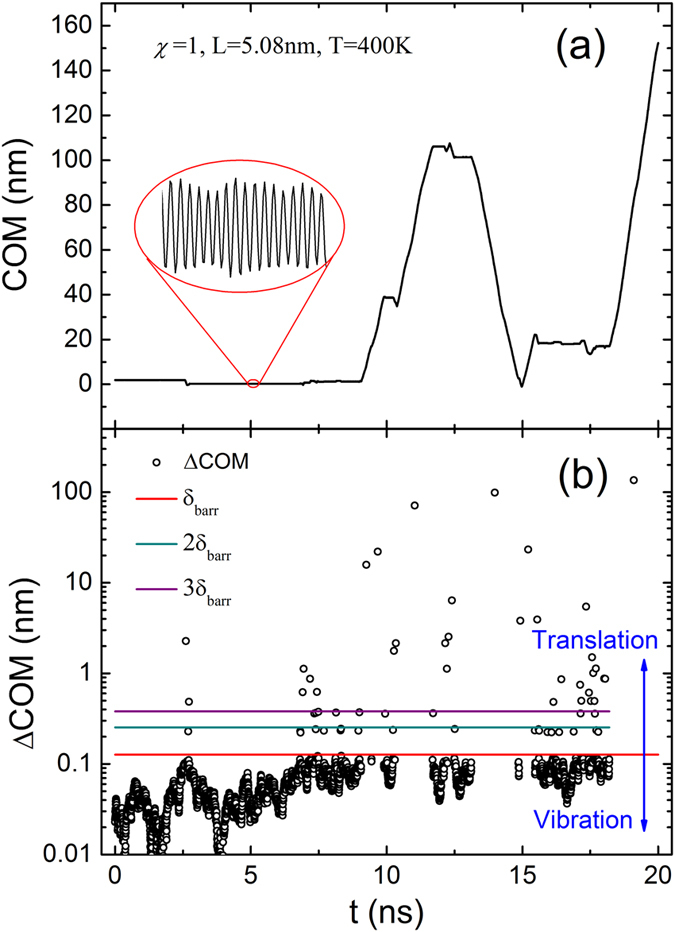
(**a**) The center of mass (COM) of a PE molecule during a 20 ns long simulation at 400 K where *χ* = 1, *L* = 5.08 nm. The PE molecule is proved to move in two motion modes: translation and vibration. (**b**) The differences of COM (ΔCOM) between the neighboring local extremums. The critical value of ΔCOM to differentiate translation and vibration is *δ*_barr_.

Shown in Fig. 3(a) is a typical COM curve of PE molecule in longitudinal direction. Obviously, the random motion could be divided into two modes: translation and vibration. In the vibration mode, the COM oscillates about an equilibrium position as shown in enlarged view. Whereas in the translation mode, the COM increases or decreases almost linearly. The transitions and durations of each motion mode are stochastic. To quantify the motion modes, the differences of COM (ΔCOM) between the neighboring local extremums of COM curve are shown in Fig. 3(b). It can be seen that the ΔCOM is stratified according to the multiples of *δ*_barr_, and the critical value to differentiate the translation and vibration modes is *δ*_barr_. Hence, the two motion modes can be extracted quantitatively to analyze their individual characteristics further.

Because a PE molecule is identical with a fluid particle that follows the Maxwell-Boltzmann (M-B) equilibrium energy distribution function. By use of the equipartition energy principle, the longitudinal mode has a kinetic energy (*E*_k_) equal to *k*_B_*T*/2 statistically, corresponding to the most probable energy distribution. Moreover, *E*_k_, the motivation influenced by *T*, fluctuates with the instantaneous interaction between atoms of PE molecule and SSWCNT; while *E*_total_, the obstruction, is determined by *χ* and *L* (or *n*_H_). Their relative values determine the motion states of PE molecule, translation (*E*_k_ > *E*_total_) or vibration (*E*_k_ < *E*_total_).

**Figure 4:**
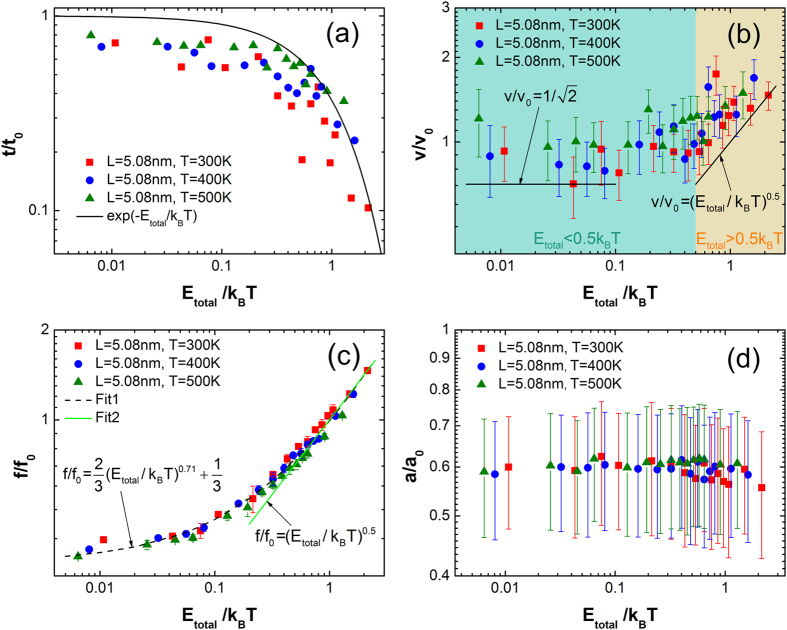
The motion characteristics versus the dimensionless activation barrier (*E*_total_/*k*_B_*T*) by controlling *χ* at 300 K, 400 K and 500 K. (**a**,**b**) are the dimensionless time (*t*/*t*_0_) and the dimensionless velocity (*v*/*v*_0_) of translation. (**c**,**d**) the dimensionless frequency (*f*/*f*_0_) and the dimensionless amplitude (*a*/*a*_0_) of vibration.

Figure 4 shows the motion characteristics of translation and vibration with respect to dimensionless activation barrier (*E*_total_/*k*_B_*T*). The results are extracted from the COM data of more than 180 cases where *L* is fixed as 5.08 nm. The data are obtained at 300 K, 400 K and 500 K with variable *E*_total_ by regulating *χ*. The values of *χ* are 0.1, 0.4, 0.7, 1, 2, 3, 4, 5, 6, 7, 8, 9, 10, 14 and 20. They are non-dimensionlized by the total simulation time (*t*_0_), 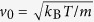, 
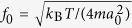
 and *a*_0_ = 1.27 Å, respectively. The results at different temperatures show a uniform trend, while for the same *χ* higher temperature results in larger translation fraction.

As shown in Fig. 4(a), the dimensionless time (*t*/*t*_0_) represents the translation fraction. With the increasing dimensionless barrier, the translation time decreases, and its trend agrees well with the equilibrium distribution function of M-B shown as solid line. This is because as the *E*_total_ increases, there is less probability for PE molecules to overcome the *E*_total_ to translate. On the contrary, as the temperature increases, the increasing *E*_k_ augments the translational probability. The M-B distribution of *E*_k_ explains the reason why the dimensionless translation time is consistent with the M-B distribution, which is consistent with the previous prediction^11^.

The translation happens when the *E*_k_ overcomes the *E*_total_. Due to the obstruction of *E*_total_, the actual translational kinetic energy (*E*_t_) will fluctuate between (*E*_k_−*E*_total_) and *E*_k_ according to energy conservation. We can approximate that the translational kinetic energy is


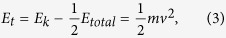


where the pre-condition for translation is *E*_k_ ≥ *E*_total_. From Eq. 3, we can get


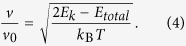


As mentioned before, the *E*_k_ is M-B distributed, and has a value equal *k*_B_*T*/2 corresponding to the most probable energy distribution. When the *E*_total_ is less than *k*_B_*T*/2, the lower bound 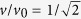 can be obtained by assign *k*_B_*T*/2 to both *E*_k_ and *E*_total_. When the *E*_total_ is greater than *k*_B_*T*/2, the lower bound 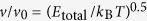 can be obtained by assign *E*_total_ to *E*_k_.

After extracting the translational sections (ΔCOM > *δ*_barr_) from the COM curve, the translational velocity can be obtained by dividing the ΔCOM by its duration. As shown in Fig. 4(b), the dimensionless velocity (*v*/*v*_0_) represents the mean translational velocity by averaging all the translation sections. Although the increasing *E*_total_ reduces translation probability, it leads to a larger translational velocity. As analyzed above, the *v*/*v*_0_ has a two-part lower bound indeed shown as solid lines, which agree well with the simulations. In the blue region (*E*_total_ < 0.5*k*_B_*T*), the *v*/*v*_0_ is almost invariant, and the velocities of lower bound are 52 m/s (300 K), 60 m/s (400 K) and 67 m/s (500 K). In the yellow region (*E*_total_ > 0.5*k*_B_*T*), the *v*/*v*_0_ increases consistently with the lower bound. It indicates that our approximations of the translational kinetic energy works well.

Shown in Fig. 4(c) is the dimensionless vibrational frequency (*f*/*f*_0_) which is the mean by averaging all the vibration sections. The *f*_0_ is on the order of a fraction of a terahertz. The dashed black line (Fit1: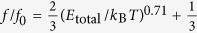) is the least squares fitting curve. The solid green line (Fit2: 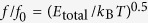) is the half of attempt frequency in a statistical point^11^. The Fit1 fits the presented data very well. However, the Fit2 only shows a good agreement when dimensionless barrier greater than 0.5. When *E*_total_/*k*_B_*T* is less than 0.5, the Fit2 seems to underestimate the *f*/*f*_0_ because of the neglect of entropy contribution caused by atomic motion, which is significant to relatively lower *E*_total_. Therefore, the current prediction (Fit2) offers good accuracy only when *E*_total_ > 0.5*k*_B_*T*. Our fitting equation (Fit1) gives a good attempt to modify the existing prediction when *E*_total_ < 0.5*k*_B_*T*.

Shown in Fig. 4(d) is the dimensionless vibrational amplitude (*a*/*a*_0_) which is the mean by averaging all the vibration sections. The amplitude shows almost unchanged when *E*_total_ < 0.5*k*_B_*T*, and decreases slightly when *E*_total_ > 0.5*k*_B_*T* at 300 K and 400 K. Because the larger barrier height of the periodical potential well will tie up the PE molecule more deeply in the potential well, resulting in a smaller vibration amplitude. This also explains the less translation probability at larger dimensionless barrier.

**Figure 5:**
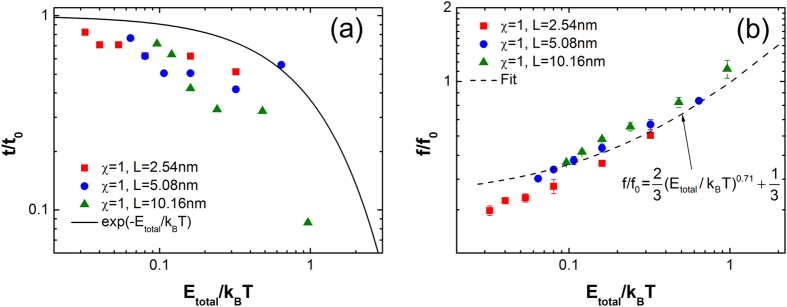
The motion characteristics versus the dimensionless activation barrier (*E*_total_/*k*_B_*T*) by controlling *T* at different lengths of PE molecules. (**a**) is the dimensionless time (*t*/*t*_0_) of translation, and (**b**) is the dimensionless frequency (*f*/*f*_0_) of vibration.

Figure 5 shows the motion characteristics for PE molecules of different *L* with respect to *E*_total_/*k*_B_*T* by regulating *T*. The different *L* results in different *m* and *E*_total_. The values of *T* are 50 K, 100 K, 200 K, 300 K, 400 K and 500 K. The dimensionless velocity and the dimensionless amplitude (not shown) shows the same trend with Fig. 4(b,d). The dimensionless time in Fig. 5(a) shows a consistent trend with the M-B distribution. However, the results are a little lower than those shown in Fig. 4(a). Moreover, the differences also can be found in Fig. 5(b), where the dashed curve (Fit: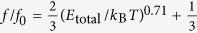) deviates from the dimensionless vibrational frequency when *E*_total_/*k*_B_*T* reduces further.

Obviously, the independent variables, *T* and *L*, are the candidate factors responsible for these differences. While the *T* can be excluded because the results in Fig. 4 shows the independence upon *T*. In addition, the results of *L* = 5.08 nm (blue) in Fig. 5(a,b) are consistent with those in Fig. 4(a,c) basically, indicating that the *L* accounts for these differences. Therefore, it is the *L* that makes the differences between the results in Figs 4 and 5. Note that, from Fig. 5(a), the shorter PE molecule tends to translate more often than the longer, because a tiny PE molecule has less *E*_total_, less inertia and larger resultant force fluctuation.

In general, the motion characteristics show great dependences on the *T*, *L* and *χ*. These three parameters are related to the *E*_k_ or *E*_total_, which determine the motion states directly. From the analysis we can conclude that small *L*, *χ* and high *T* are beneficial to the translational mode. It has guiding significance for the designs of NHP devices, where the translational mode is favorable. This predication is consistent with the previous works, where the mass transports often happen when the invading materials are liquid^4^ or the melted metals or ionic compounds at high temperature^6^,^7^,^10^,^14^.

## Part 2: Nano-Heat Pipe

### Models and Methods

**Figure 6:**
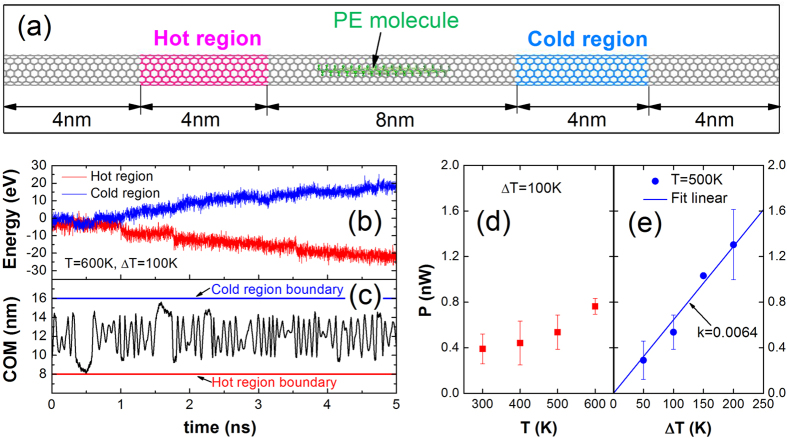
(**a**) The model of NPH composed of a (7, 7) SWCNT and a PE molecule. (**b**) The heat exchange of NHP and (**c**) the COM of the PE molecule with a temperature difference of 100 K at 600 K with respect to simulation time. (**d**) The heat exchanging power (*P*) with respect to working temperature (*T*). (**e**) The *P* with respect to the temperature difference (Δ*T*).

The random walk effect of the PE molecule in SWCNT motivates the proposition of NHP, as shown in Fig. 6(a). The SWCNT is divided into five parts. Non-equilibrium molecular dynamics simulations (NEMD) are used to calculate the heat exchange performance with a periodic boundary condition applied in the longitudinal direction. The hot region (red) and cold region (blue) are the heat source and heat sink, respectively, whose temperatures are 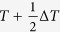 and 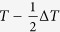. The grey parts of SWCNT are frozen, so that the PE molecule is the only carrier to convey the heat from hot to cold region.

The atomic interactions of PE molecule and SWCNT are described by an AIREBO potential. The motion equations are integrated by the Velocity Verlet algorithm with a time step of 0.2 fs. Initially, Nose-Hoover heat reservoir is used to equilibrate the system at desired temperature *T* for 200 ps. Then, the simulations run in microcanonical ensemble to eliminate the after-effects of the heat reservoir for 400 ps. After that, the Langevin thermostats are used to build a temperature difference. Finally, the heat flows and the longitudinal COMs of PE molecule are recorded for another 5 ns.

### Results and Discussions

The NHP’s heat exchanging performance is shown in Fig. 6(b–e). By NEMD, the reciprocating motion of PE molecule between hot and cold region is shown in Fig. 6(c). The COM represents the position of the PE molecule in the longitudinal direction. Due to the temperature difference between hot and cold region, the PE molecule will absorb heat from hot region atoms when arriving hot region, and release heat to cold region atoms when arriving cold region. During the heat exchange process, the PE molecule acts as a carriage; the heat acts as the cargo; and the random walk effect is the driven power. The heat exchange borne by this reciprocating motion is shown in Fig. 6(b). The energy added to hot region (red) and subtracted to cold region (blue) is the same.

The Fig. 6(d,e) show the heat exchanging power (*P*), which are the average slopes of heat exchange curves (red and blue) in Fig. 6(b). As shown in Fig. 6(d), when the Δ*T* is fixed as 100 K, the *P* increases with the increasing *T* from 300 K to 600 K. As shown in Fig. 6(e), when the *T* is fixed as 500 K, the *P* increases with the increasing Δ*T* from 50 K to 200 K. Hence, the larger *T* and Δ*T* will lead to a larger *P*. When T = 500 K, by assembling NHP arrays where the quantity density is 7 × 10^13^ cm^−2^, the heat exchanging coefficient can reach as high as 450 WK^−1^ cm^−2^ according to the linear fitting in Fig. 6(e). If with any optimizations, a higher heat exchanging coefficient can be achieved readily. Note that this heat exchanging performance doesn’t include the ability of SWCNTs. These NHP devices with high heat exchanging performance are qualified to applications in nanoscale hotspots and thermal interfaces potentially.

### Conclusions

In summary, by EMD simulations, we analyze the motions mechanisms of the random walk effect employing a SSWCNT. The results show that the motion characteristics show great dependences on the *T*, *L* and *χ* that small *L*, *χ* and high *T* are beneficial to the translational mode. Our analyses have shown some new motion characteristics that previous predictions have never had. The differences from previous predictions greatly enrich the mass transport theory of random walk theory. Further, we propose a novel NHP based on the findings. And NEMD simulations are used to study heat exchanging performance of NHP, which can reach as high as 450 WK^−1^ cm^−2^ at 500 K by assembling NHP arrays with a quantity density of 7 × 10^13^ cm^−2^. These NHP devices are potential to be applied in thermal interfaces and nanoscale thermal regulations. This study will benefit the designs of NHP and other nanoscale mass transport devices.

## Additional Information

**How to cite this article**: Liao, Q. *et al*. Spontaneous Migration of Polyethylene Molecule Sheathed inside Single-Walled Carbon Nanotube for Nano-Heat Pipe. *Sci. Rep*. **6**, 26441; doi: 10.1038/srep26441 (2016).

## Supplementary Material

Supplementary Information

Supplementary Movie S1

Supplementary Movie S2
