# Supplementary material for: Spontaneous Migration of Polyethylene Molecule Sheathed inside Single-Walled Carbon Nanotube for Nano-Heat Pipe
Source: Sci Rep. 2016 May 23;6:26441. doi: 10.1038/srep26441 (PMC4876425; doi:10.1038/srep26441)
Supplement: Supplementary Information [file srep26441-s1.doc]

Supporting information

**Spontaneous Migration of Polyethylene Molecule Sheathed inside Single-Walled Carbon Nanotube for Nano-Heat Pipe**

Quanwen Liao, Zhichun Liu^+^, Nuo Yang, Wei Liu^+^

School of Energy and Power Engineering, Huazhong University of Science and Technology (HUST), Wuhan 430074, People's Republic of China

^+^Corresponding authors: Z.L. ([zcliu@hust.edu.cn](mailto:zcliu@hust.edu.cn)), W.L. ([w_liu@hust.edu.cn](mailto:w_liu@hust.edu.cn))

**Videos of spontaneous migration**

To illustrate spontaneous migration clearly, we provide several animate Gif videos about the equilibrium molecular dynamics (EMD) simulations. In EMD simulations, simplified single-walled carbon nanotube (SSWCNT) is applied. This spontaneous migration can be seen in animate Gif S1, where the SSWCNT outside the PE is not shown. The animate Gif S2 shows a spontaneous migration of PE molecule inside SSWCNT. This phenomenon of spontaneous migration is very well reproduced in both animate Gif S1 and animate Gif S2, where EMD simulations are conducted at 300K with periodic boundary condition in longitudinal direction. The Fig. S1 is the cross-section projection of animate Gif S1, we can see that the cross-sectional motions of PE’s atoms is confined very well by using SSWCNT. It shows the SSWCNT works effectively.

Gif S1 The spontaneous migration of PE, the SSWCNT outside the PE is not shown.

Gif S2 The spontaneous migration of PE molecule inside SSWCNT, the SSWCNT outside is not shown.


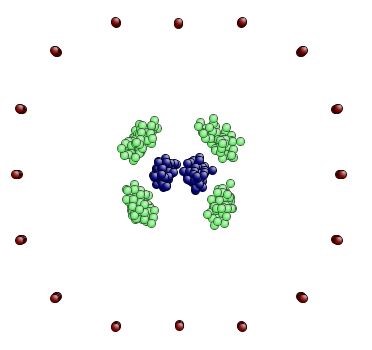


Fig. S1 The cross-section projection of animate Gif S1. The outside atoms denote SSWCNT. The inside atoms denote PE.
