# Supplementary figures and images for: Spontaneous Migration of Polyethylene Molecule Sheathed inside Single-Walled Carbon Nanotube for Nano-Heat Pipe
Source: Sci Rep. 2016 May 23;6:26441. doi: 10.1038/srep26441 (PMC4876425; doi:10.1038/srep26441)

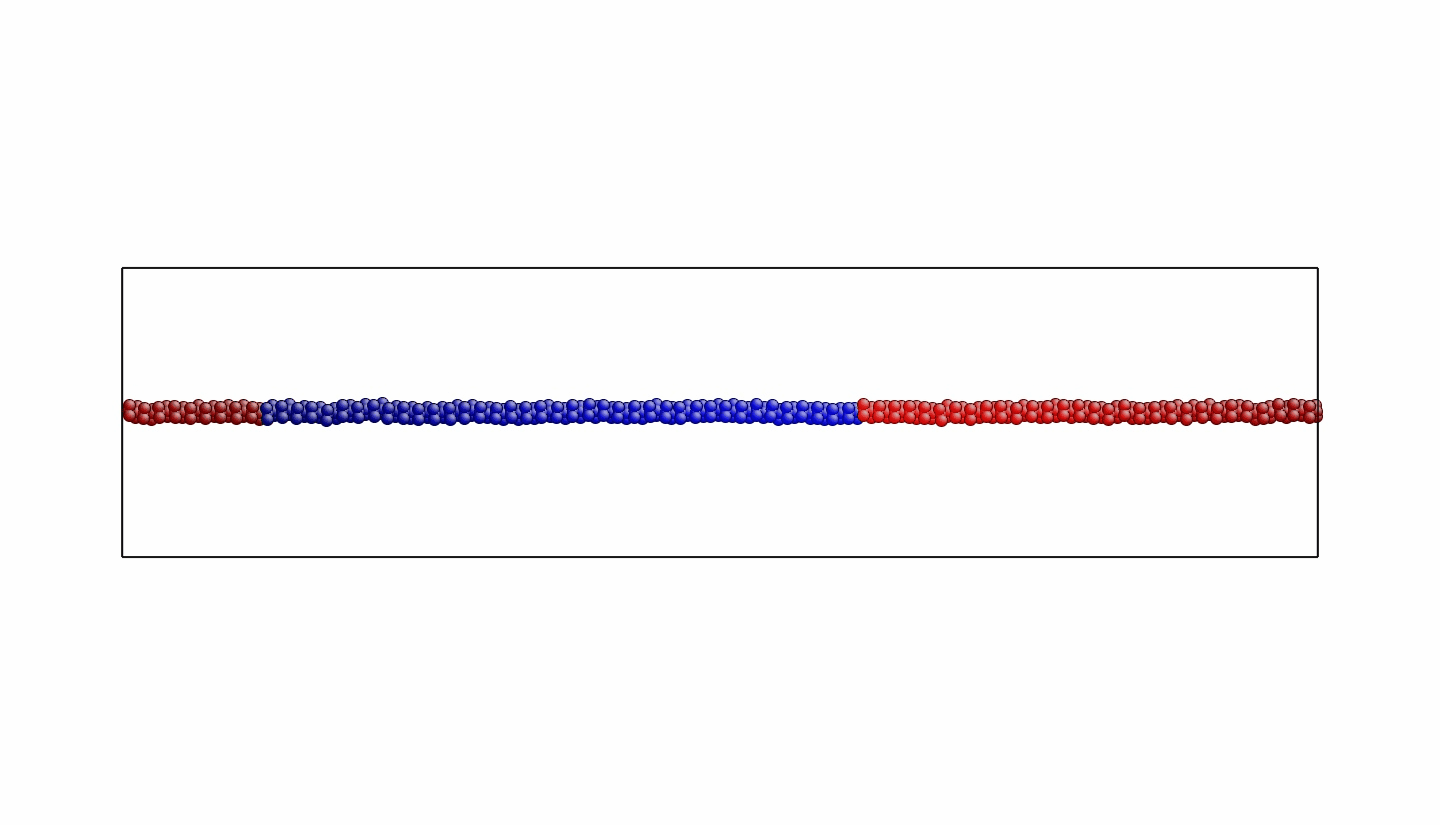

Supplement: Supplementary Movie S1 [file srep26441-s2.gif]

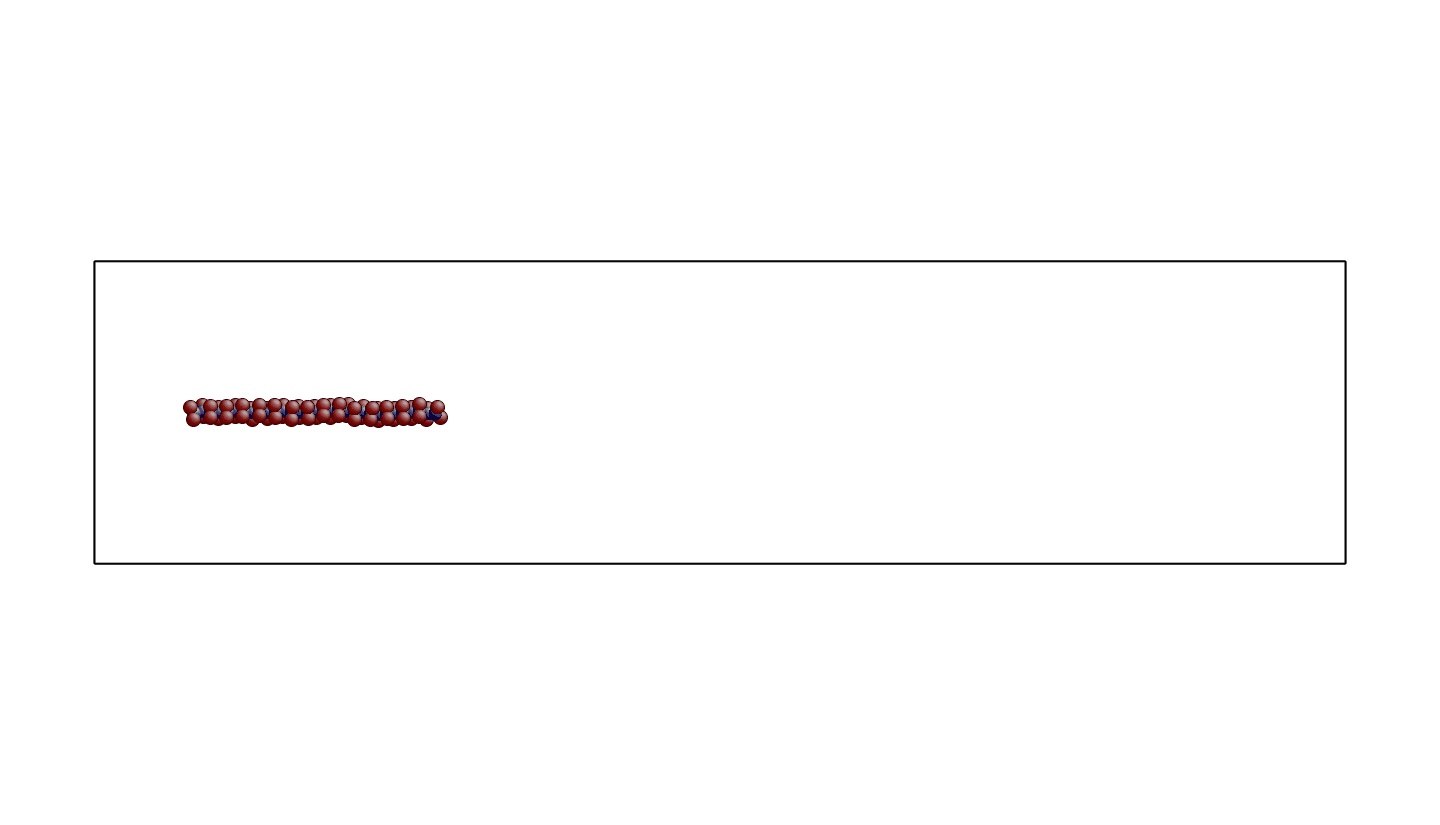

Supplement: Supplementary Movie S2 [file srep26441-s3.gif]
